# Assessing causality between different risk factors and pulmonary embolism: A Mendelian randomization study

**DOI:** 10.3389/fcvm.2023.1057019

**Published:** 2023-02-23

**Authors:** Jian-ming Wei, Yan-li Song, Huan Zeng, Wen-wen Yan, Xue-bo Liu

**Affiliations:** ^1^Department of Emergency Medicine, Shanghai Tongji Hospital, Tongji University School of Medicine, Shanghai, China; ^2^Department of Cardiology, Shanghai Tongji Hospital, Tongji University School of Medicine, Shanghai, China

**Keywords:** body mass index, ever smoked, heart failure, alcohol intake frequency, inflammatory bowel disease, pulmonary embolism, Mendelian randomization

## Abstract

**Objectives:**

Mendelian randomization (MR) was used to estimate the causal relationship between body mass index (BMI), ever smoked, heart failure, alcohol intake frequency, inflammatory bowel disease (IBD), and pulmonary embolism (PE). This study aimed to investigate whether there is a causal relationship between BMI, the presence of smoking, heart failure, frequency of alcohol intake, IBD, and PE.

**Methods:**

Pooled data on PE from a published GWAS meta-analysis involving approximately 461,164 participants of European ancestry were selected. A publicly available pooled dataset of BMI (461,460), ever smokers (461,066), heart failure (977,323), IBD (75,000), and frequency of alcohol intake (462,346) was used from another independent GWAS. MR was performed using established analysis methods, including Wald ratios, inverse variance weighted (IVW), weighted median (WM), and MR-Egger. Also, the final expansion was validated with multivariate MR.

**Results:**

In the IVW model, genetically elevated BMI was causally associated with PE [OR = 1.002, 95% CI (1.001, 1004), *P* = 0.039]. Cochran’s *Q* test was used to detect heterogeneity in the MR-Egger analysis (*P* = 0.576). Therefore, the effect of gene-level heterogeneity was not considered. In the MR analysis of other risk factors, we observed genes for ever smoking [IVW OR = 1.004, 95% CI (0.997, 1.012)], heart failure [IVW OR = 0.999, 95% CI (0.996, 1.001)], IBD [IVW OR = 1.000, 95% CI (0.999, 1.001)], and frequency of alcohol intake [IVW OR = 1.002, 95% CI (1.000, 1.004)] were not causally associated with PE. Analysis using multivariate MR expansion showed no causal effect of BMI on PE considering the effect of height as well as weight (*P* = 0.926).

**Conclusion:**

In European populations, a causal relationship exists between BMI and PE: increased BMI leads to PE. In contrast, ever smoking, heart failure, frequency of alcohol intake, and IBD are not directly associated with PE. There was no causal effect of BMI with PE in multivariate Mendelian randomized analysis.

## Introduction

Venous thromboembolism (VTE), including deep vein thrombosis (DVT) and pulmonary embolism (PE), affects nearly 10 million people of all ethnicity worldwide each year (1). In Europe, 8–13 per 1,000 women aged 15–55 years and 2–7 per 1,000 men die from PE (2). Therefore, early identification and active intervention of risk factors in patients with PE is essential in clinical practice. There is a need to explore the etiology and prevent the occurrence of PE.

Venous thromboembolism is a common venous thrombotic event, and its common risk factors include ever smoking and obesity (3–5). In addition, heart failure has been reported to increase the risk of PE (6). Several studies have shown that the risk of VTE is 2–3 times higher in patients with inflammatory bowel disease (IBD) than in the general population (7). Moderate frequency of alcohol intake has been differentially associated with hemostasis and fibrinolytic factor levels (8). However, the relationship between the frequency of alcohol intake and the risk of developing PE remains uncertain.

Previous literature has suggested that risk factors for PE include body mass index (BMI), ever smoking, heart failure, IBD, and frequency of alcohol intake. Although there is considerable evidence to support the association of these risk factors with PE, the direction of causality between these risk factors and PE remains unclear (9). Due to the influence of confounding factors, the available clinical findings do not directly imply a causal relationship. MR methods were subsequently introduced to reduce the impact of acquired confounding factors (10).

Mendelian randomization (MR) is a powerful tool in epidemiology to estimate the causal effect of exposure on outcome in the presence of unobserved confounders by exploiting genetic variation as instrumental variables (IVs) of exposure (11). Nowadays, causality based on the MR of inferred variables is widely used. In this MR analysis, we aimed to demonstrate whether there is a causal relationship between BMI, ever smoking, heart failure, frequency of alcohol intake, IBD, and PE.

## Materials and methods

### Overview of the study design

**FIGURE 1:**
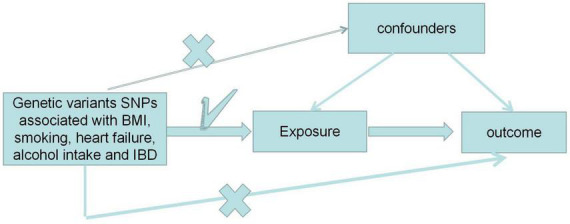
Design of the Mendelian randomization (MR) study. “X” indicates that the genetic variant is not associated with confounding factors or cannot be directly involved in the outcome through the exposure pathway. “√” means that the genetic variant is highly correlated with exposure. SNP, single nucleotide polymorphism.

An overview of the multifactorial MR analysis study is shown in Figure 1. In summary, we first assessed the causal effects of BMI, ever smoking, heart failure, frequency of alcohol intake, and IBD on PE. Also, genetic variation was considered IV only if three strict assumptions were met (12). First, genetic variation was highly correlated with exposure. Second, genetic variants are independent of confounding factors (e.g., BMI, sex, and age). Finally, genetic variation directly affects the outcome through the exposure pathway. We analyzed this using a recently pooled dataset of genome-wide association studies (GWAS) on BMI, ever smoked, heart failure, frequency of alcohol intake, and IBD on PE.

### Data sources and SNP selection for BMI, ever smoked, heart failure, frequency of alcohol intake, and IBD

We selected pooled data for PE from published GWAS meta-analyses involving approximately 461,164 participants of European ancestry. We also used a publicly available pooled dataset of BMI (including 461,460), ever smoking (including 46,106), heart failure (including 977,323), IBD (including 75,000), and alcohol intake frequency (including 462,346) from another independent GWAS. The GWAS reported data related to BMI, ever smoking, heart failure, IBD, and alcohol intake frequency with genome-wide significance at the PE level (*P* < 5 × 10^–8^). Also, these single nucleotide polymorphisms (SNPs) were tested for linkage disequilibrium (LD) to cluster the independence of SNPs. Genome-wide significant independent (*r*^2^ < 0.001) variants associated with BMI, ever smoking, heart failure, IBD, and alcohol intake frequency were extracted from the GWAS. In addition, *R*^2^ and *F* statistics were calculated to assess the strength of IVs based on the sample size of the exposure dataset, the number of IVs, and genetic variance. Finally, we searched the Phenoscanner database^1^ for all exposure-associated SNPs and their proxies to determine if there were SNPs associated with confounders (*P* < 5 × 10^–8^). We manually removed these SNPs to avoid polymorphic effects.

### MR analysis

We used the two-sample MR package in the R language for the analysis. We mainly used inverse variance weighted (IVW) for MR analysis, and predictions were made by weighted regression of SNP-specific Wald ratios (i.e., β outcome/β exposure). Several sensitivity analyses were performed, including weighted median (WM) and MR-Egger regression methods. IVW estimates are inverse variance-weighted means of ratio estimates from two or more instruments (13). The WM estimate is the median of the weighted empirical distribution function of the individual SNP ratio estimates. MR-Egger regression consists of a weighted linear regression of SNP schizophrenia on SNP biomarker effect estimates (14). The MR-Egger regression method is robust to horizontal pleiotropy. We quantified the level of heterogeneity by using Cochran’s *Q* statistics and *I*^2^ statistics (15). The larger the value of *I*^2^ was, the more significant the heterogeneity. In addition, the “leave-one-out analysis,” which removed each SNP in turn, ensured the reliability of the results.

## Results

### Causal effects of BMI, ever smoking, heart failure, frequency of alcohol intake, and IBD on PE

**FIGURE 2:**
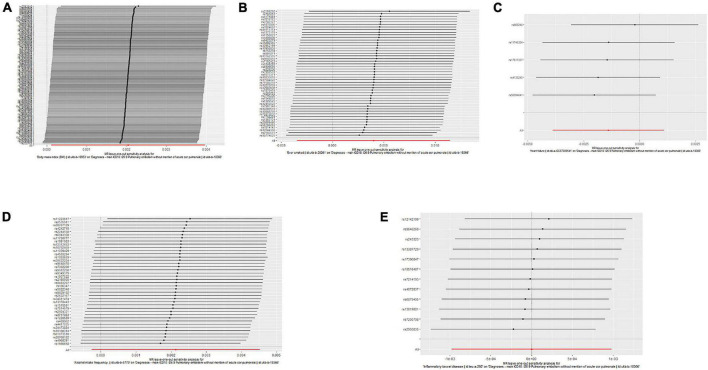
“Leave-one-out” analysis shows the effects of body mass index (BMI), ever smoking, heart failure, frequency of alcohol intake, and inflammatory bowel disease (IBD)-related single nucleotide polymorphism (SNPs) on pulmonary embolism (PE). Mendelian randomization (MR) estimated effect sizes for BMI **(A)**, ever smoking **(B)**, heart failure **(C)**, frequency of alcohol intake **(D)**, and IBD **(E)** are shown. Data are expressed as β values and 95% CI.

**FIGURE 3:**
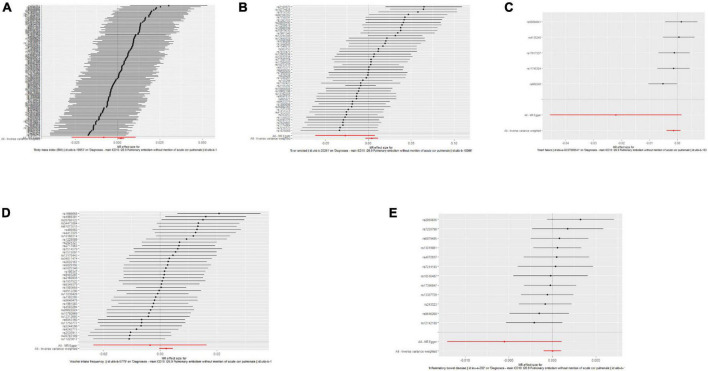
Forest plots of the causal effects of body mass index (BMI), ever-smoking, heart failure, alcohol intake frequency, and inflammatory bowel disease (IBD)-related single nucleotide polymorphism (SNPs) on pulmonary embolism (PE). The Mendelian randomization (MR) estimated effect sizes for BMI **(A)**, ever smoking **(B)**, heart failure **(C)**, frequency of alcohol intake **(D)**, and IBD **(E)** are shown. Data are expressed as beta values and 95% CI.

**FIGURE 4:**
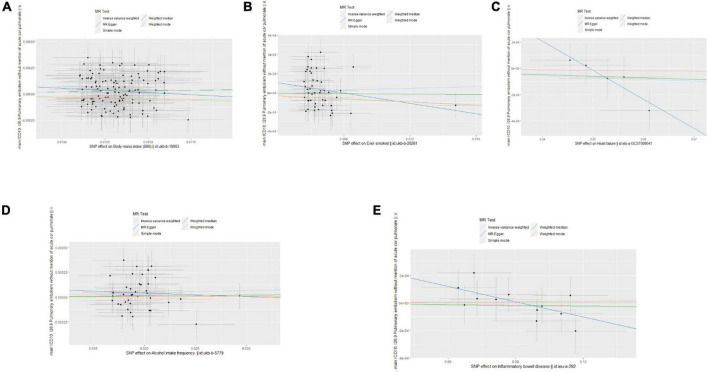
Scatter plots of the causal effects of body mass index (BMI), ever smoking, heart failure, alcohol intake frequency, and inflammatory bowel disease (IBD)-related single nucleotide polymorphism (SNPs) on pulmonary embolism (PE). The effect sizes of Mendelian randomization (MR) estimates for BMI **(A)**, ever smoking **(B)**, heart failure **(C)**, frequency of alcohol intake **(D)**, and IBD **(E)** are shown.

**FIGURE 5:**
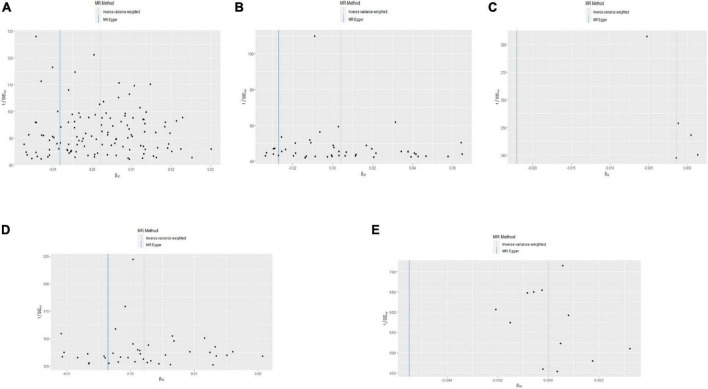
Funnel plot of the causal effects of body mass index (BMI), ever smoking, heart failure, alcohol intake frequency, and inflammatory bowel disease (IBD)-related single nucleotide polymorphism (SNPs) on pulmonary embolism (PE). The Mendelian randomization (MR) estimated effect sizes for BMI **(A)**, ever smoking **(B)**, heart failure **(C)**, alcohol intake frequency **(D)**, and IBD **(E)** are shown.

Single nucleotide polymorphisms associated with BMI, ever smoking, heart failure, frequency of alcohol intake, and IBD were collected and downloaded from the phenotype database. Confounders were controlled using (see text footnote 1), and SNPs directly associated with weight, height, BMI, smoking, heart failure, alcohol intake, and IBD were excluded. SNPs associated with outcome PE were also excluded. In addition, 172, 57, 10, 71, and 21 genetic variants not associated with LD were identified as IVs, respectively. To verify the influence of each SNP on the overall causal estimate, leave-one-out analysis was performed (as shown in Figure 2). Forest plot shows the odds ratio (OR) with a horizontal line representing 95% CI for the BMI, ever smoking, heart failure, frequency of alcohol intake, and IBD -associated SNP allele for PE risk (as shown in Figure 3). Scatter plot to visualize causal effect of BMI, ever smoking, heart failure, alcohol intake frequency, and IBD on total PE risk (as shown in Figure 4). Funnel plots to visualize overall heterogeneity of MR estimates for the effect of BMI, ever smoking, heart failure, alcohol intake frequency, and IBD on PE randomization (as shown in Figure 5). The F-statistics of IVs for BMI, ever smoking, heart failure, alcohol intake frequency, and IBD were above the threshold of 10, indicating that IVs were vital instruments, thus reducing bias in IV estimation.

**FIGURE 6:**
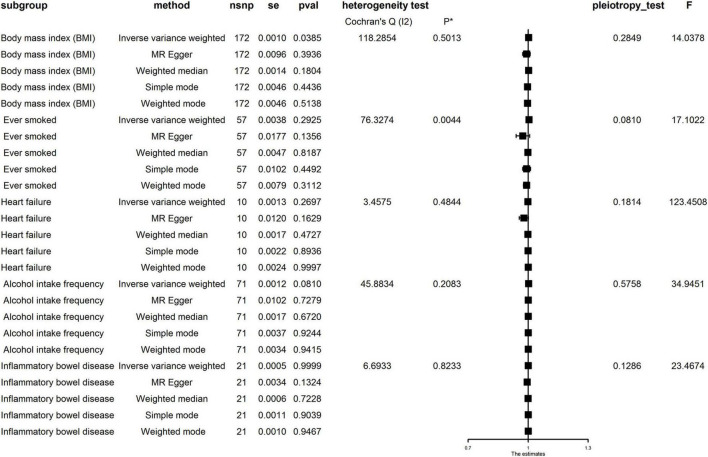
Mendelian randomization (MR) analysis for the causality of body mass index (BMI), ever smoking, heart failure, alcohol intake frequency, and inflammatory bowel disease (IBD) with the risk of pulmonary embolism (PE). MR, Mendelian randomization; IVW, inverse variance weighted; OR, odds ratio; CI, confidence interval; WM, weighted median.

As shown in Figure 6, pleiotropy was detected by *P* for Intercept (*P* > 0.05). Therefore, we used IVW in the random effects model. Hereditarily elevated BMI was causally associated with PE in the IVW model (OR = 1.002, 95% CI: 1.001–1004, *P* = 0.039). Heterogeneity was detected by MR-Egger analysis with Cochran’s *Q* test (*P* = 0.576). Therefore, the effect of gene-level heterogeneity was not considered.

In the MR analysis of other risk factors, we observed genes for ever smoking (IVW OR = 1.004, 95% CI: 0.997–1.012), heart failure (IVWOR = 0.999, 95% CI: 0.996–1.001), IBD (IVWOR = 1.000, 95% CI: 0.999–1.001), and alcohol intake frequency (IVW OR = 1.002, 95% CI: 1.000–1.004) levels were not causally related to PE. Cochran’s *Q* test showed heterogeneity only in the case of ever smoking but not in other factors. In addition, MR-Egger analysis showed non-directional pleiotropy in all directions for IVs (*P* for Intercept = 0.081 for ever smoking, *P* for Intercept = 0.181 for heart failure, *P* for Intercept = 0.126 for IBD, and *P* for Intercept = 0.285 for frequency of alcohol intake). Analysis using multivariate MR expansion showed no causal effect of BMI on PE considering the effect of height as well as weight (*P* = 0.926).

## Discussion

This study used MRs for BMI, ever smoking, heart failure, alcohol intake frequency, and IBD from the available GWAS database to infer a causal relationship between BMI and PE. However, there was no causal relationship between ever smoking, heart failure, alcohol intake frequency, and IBD and PE. It was observed that ever smoking, heart failure, IBD, and frequency of alcohol intake could not directly cause PE at the genetic level.

A systematic review and dose-response meta-analysis of 3910747 participants showed a significant association between lower BMI (underweight *vs.* normal BMI) and a reduced risk of PE (HR: 0.80, 95% CI: 0.70–0.92, *I*^2^ = 9%) and a higher risk of PE in obese than those participants presenting with a healthy BMI (HR: 2.24, 95% CI: 1.93–2.60, *I*^2^ = 0%) (16). Another MR study used BMI-related SNPs from the UK Biobank as an instrumental variable to assess the association between 367,703 participants with cardiovascular disease. It was concluded that BMI was positively associated with PE. The OR for PE was 1.06 (95% CI: 1.02–1.11; *P* = 2.6 × 10^–3^) for every 1 kg/m^2^ increase in BMI (9). However, the study did not observe a different causal relationship for PE.

At the molecular level, high-throughput proteomics and cross-validated regularized regression modeling approaches showed 11 proteins (CLEC4C, FABP4, FLT3LG, IL-17G, LEP, LYVE1MASP1, ST2, THBS2, THBS4, and TSLP) were consistently associated with BMI in plasma in the context of acute VTE (17–27). Furthermore, the absence of serum leptin is inversely associated with recurrence and death from VTE at high body weight or high circulating concentrations of MMP-2 (28). Whether the body influences VTE through the regulation of leptin remains to be further refined.

This study has several limitations. First, the results of this study were only set in a European population, and extrapolation to other populations still requires further validation. Second, the findings were not stratified by BMI, and the effect of different BMI strata on PE could not be further clarified. Third, some risk factors involved several SNPs. Therefore, a larger GWAS and more SNPs are needed as tools to replicate the MR study to improve the ability to test associations. More importantly, the MR design still needs to be validated by RCT.

Nevertheless, the present study undoubtedly has some advantages. First, it involved a large cohort, enabling multifactorial causal inference of an outcome event in a real-world study. Because the effects of confounding factors were controlled for and tested by various methods, the conclusion obtained had a solid evidence base and could better guide clinical practice. Second, the study population was PE, a common cardiovascular disease with high annual morbidity and mortality. This study explored the effects of five common factors, namely, BMI, ever smoking, heart failure, frequency of alcohol intake, and IBD, on PE with good generalizability. These five factors are common in current clinical practice.

Although existing clinical studies have shown that previous smoking, heart failure, frequency of alcohol consumption, and IBD are associated with PE, there was no causal effect between the above risk factors and PE using single-factor MR analysis, and no causal effect between BMI and PE in the multifactor MR extension analysis, corrected for height and weight. The possible reasons for this are as follows: (1) whether there are other different mediators mediating the association between the above risk factors and PE, but considering the limited data obtained in this study, further expansion of the analysis of the mediators of the above risk factors cannot be supported for the time being; (2) the data from the existing study are limited to the European population, and the expansion of this conclusion needs to consider the heterogeneity of different populations, and more data are needed to verify the causal effect of the above risk factors and PE in the future. In the future, more data are needed to verify the causal effects of the above risk factors and PE; meanwhile, the above findings need to be further validated by RCT. There are no clinical studies available that suggest that the association between PE and ever smoking, heart failure, frequency of alcohol consumption, or IBD is mediated by other intermediate risk factors, which is something that needs to be further explored in the future in this study. (3) Restricted by the number of SNPs as well as the *F*-value, the higher the number of SNPs and the larger the *F*-value, the more statistically significant the effect.

In conclusion, there was a causal relationship between BMI and PE in the European population. Increased BMI could lead to PE, while the presence or absence of smoking, heart failure, frequency of alcohol intake, and IBD are not directly causally related to PE. There was no causal effect of BMI with PE in multivariate Mendelian randomized analysis. Either univariate or multivariate MR needs to be further validated by RCT in the future.

## Data availability statement

The original contributions presented in this study are included in the article/Supplementary material, further inquiries can be directed to the corresponding authors.

## Author contributions

J-MW, Y-LS, and HZ performed the material preparation, data collection, and analysis. J-MW, W-WY, and X-BL wrote the first draft of the manuscript. W-WY and X-BL commented on previous versions of the manuscript. All authors contributed to the study conception and design and read, revised, and approved the final manuscript.

## Footnotes

1
http://www.phenoscanner.medschl.cam.ac.uk/
