# Supplementary figures and images for: Assessing causality between different risk factors and pulmonary embolism: A Mendelian randomization study
Source: Front Cardiovasc Med. 2023 Feb 23;10:1057019. doi: 10.3389/fcvm.2023.1057019 (PMC9996005; doi:10.3389/fcvm.2023.1057019)

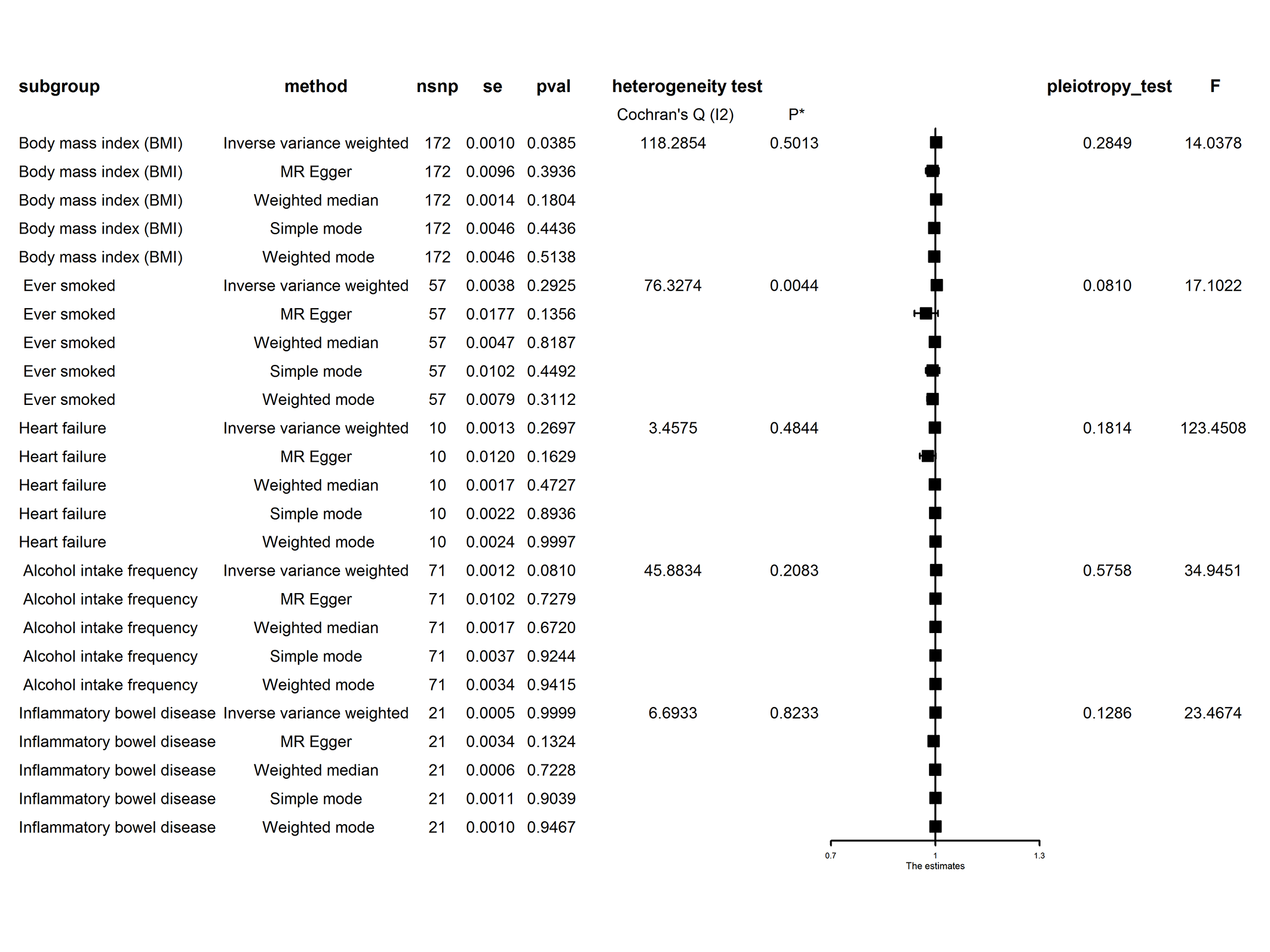

Supplement: Supplementary file 2 [file Data_Sheet_2.ZIP › 6.tif]

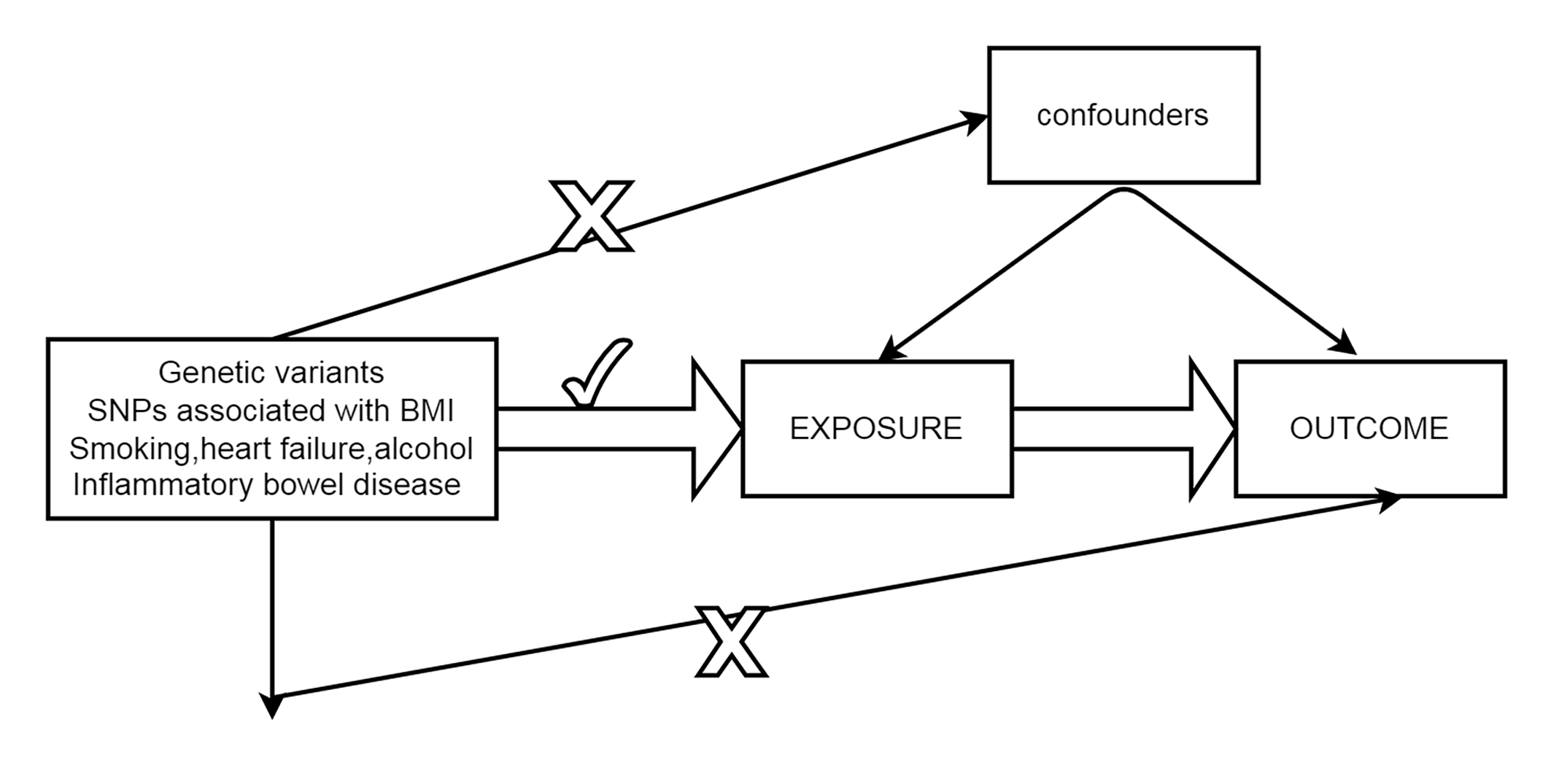

Supplement: Supplementary file 2 [file Data_Sheet_2.ZIP › 1.png]

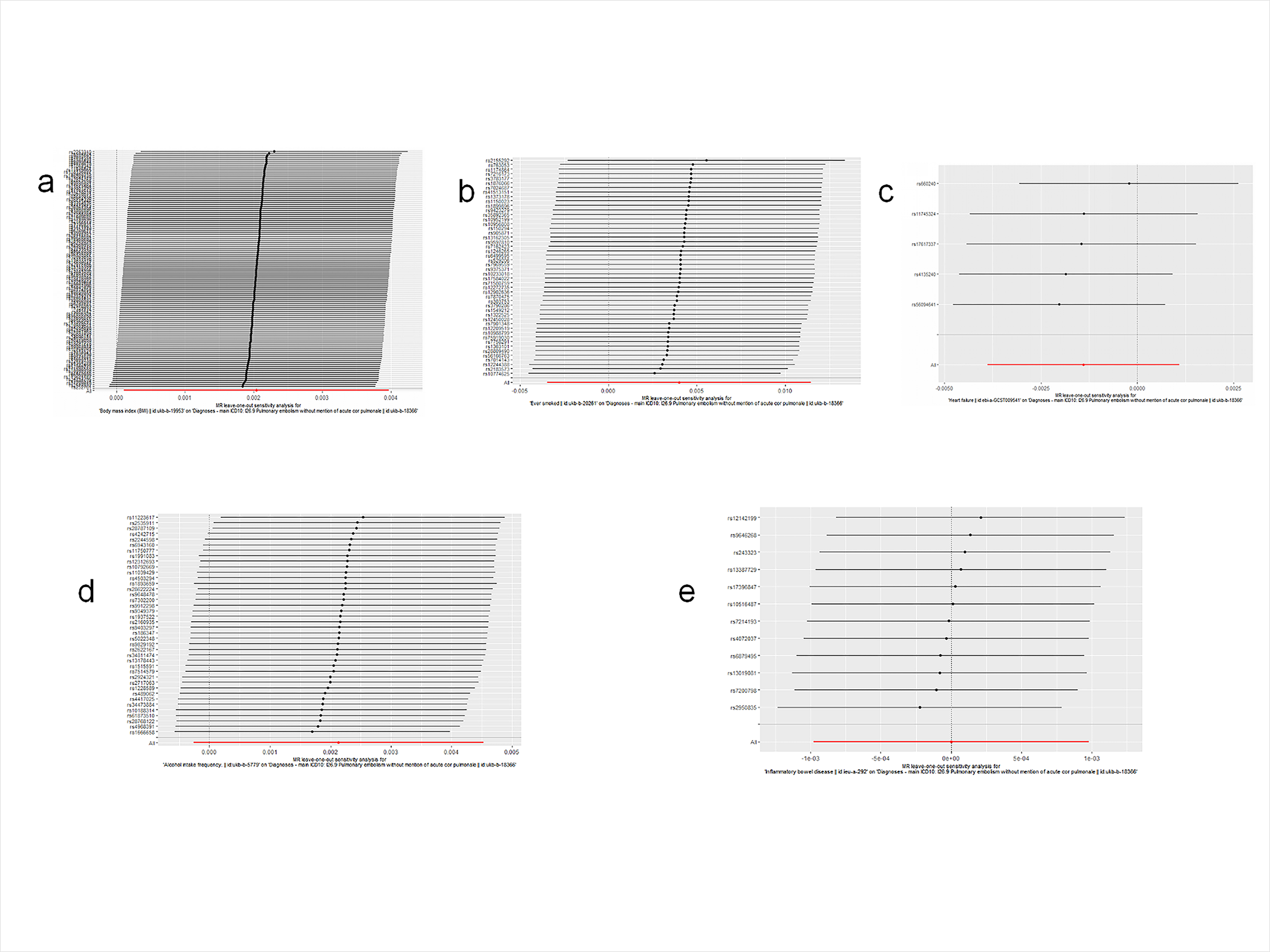

Supplement: Supplementary file 2 [file Data_Sheet_2.ZIP › 2.tif]

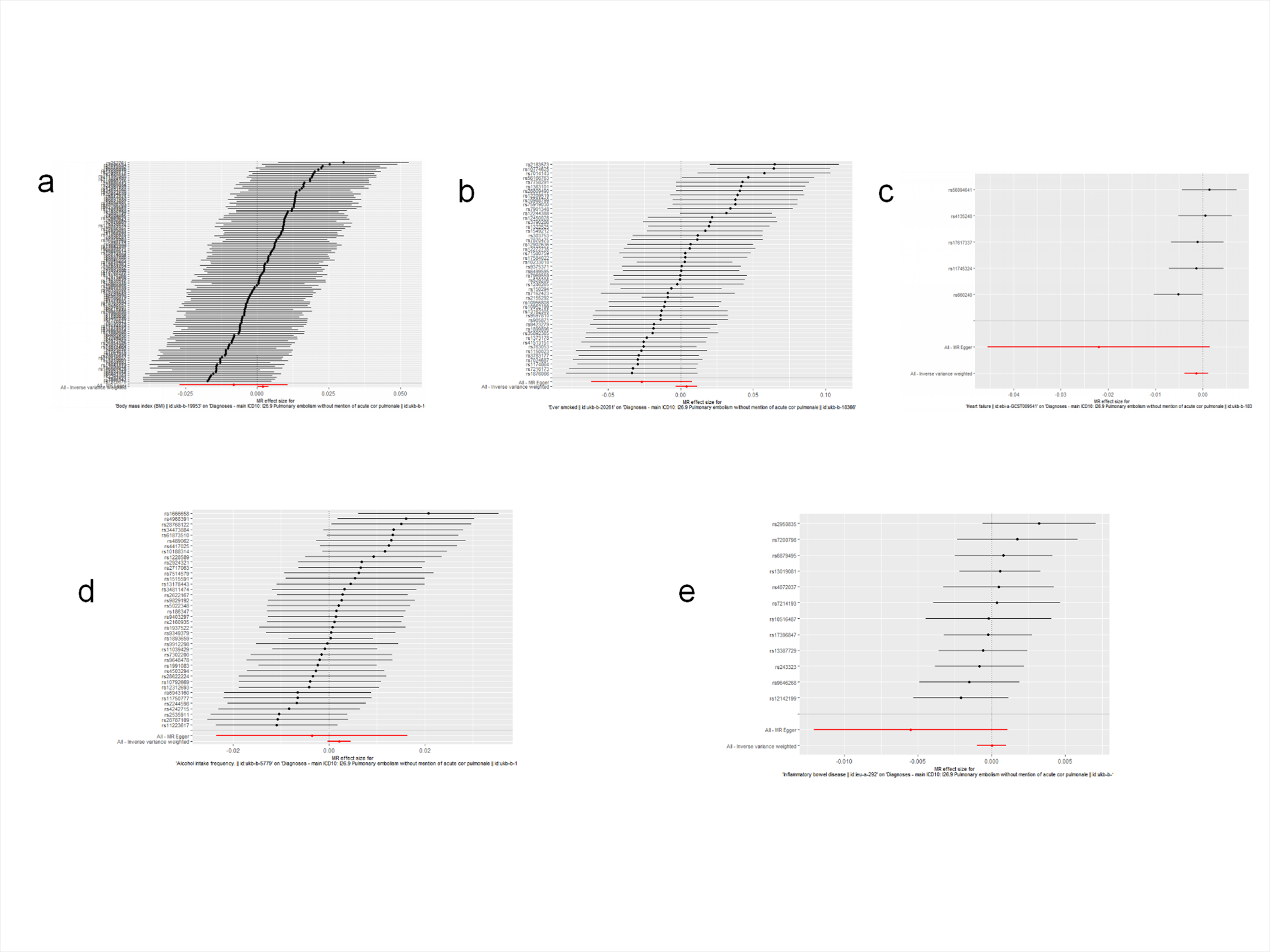

Supplement: Supplementary file 2 [file Data_Sheet_2.ZIP › 3.tif]

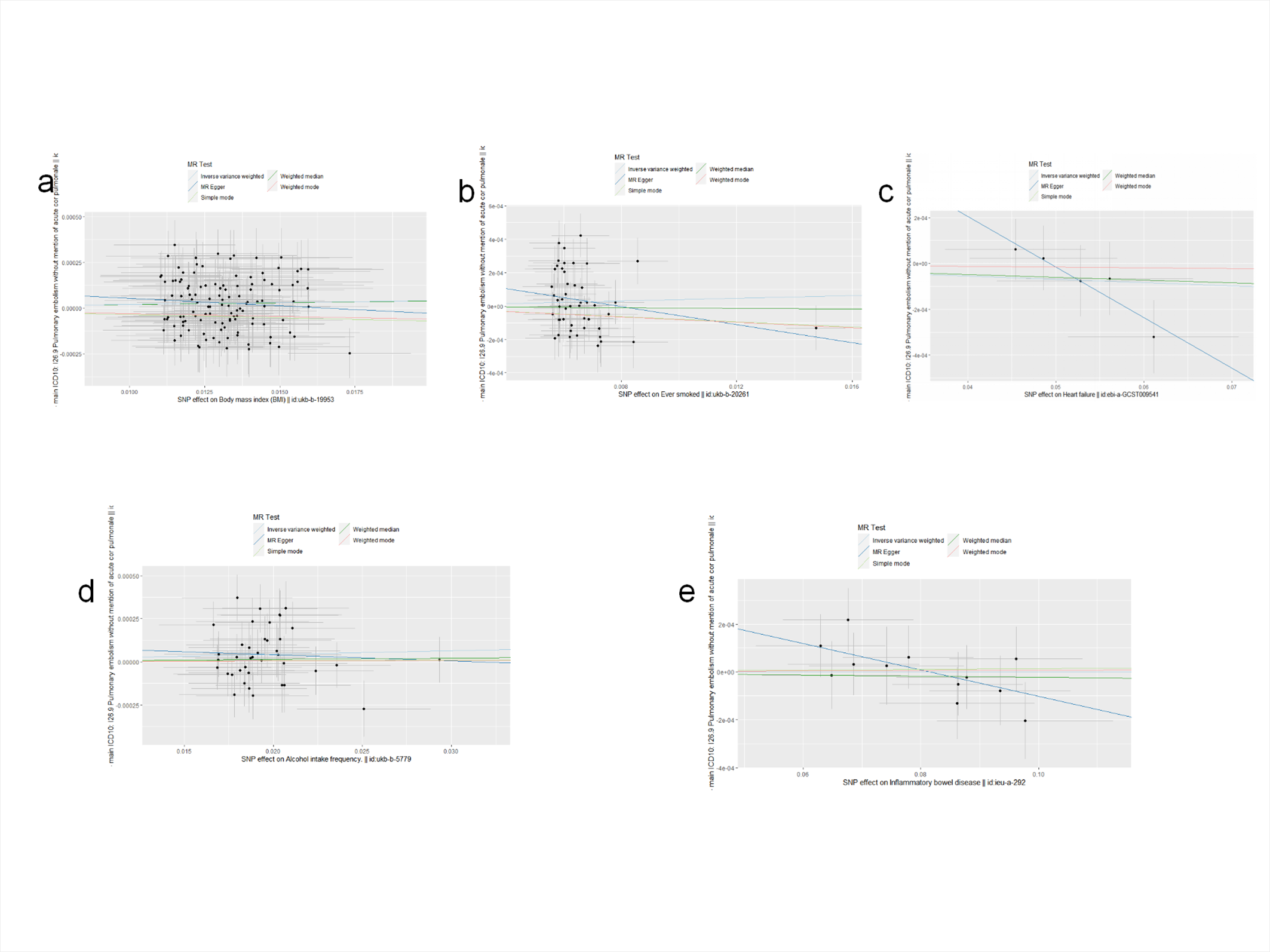

Supplement: Supplementary file 2 [file Data_Sheet_2.ZIP › 4.tif]

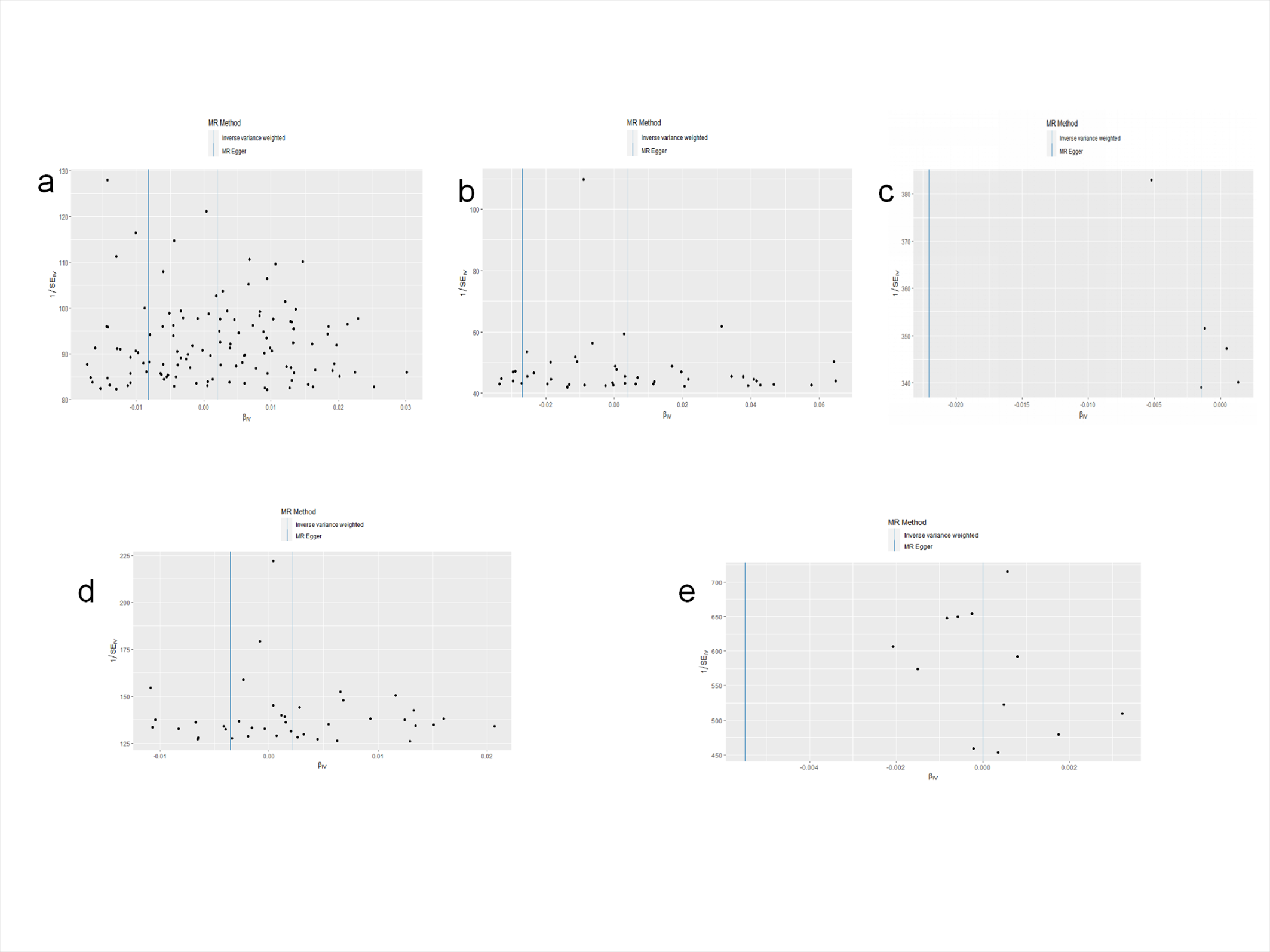

Supplement: Supplementary file 2 [file Data_Sheet_2.ZIP › 5.tif]
